# Incidence and predictors of lost to follow-up among drug-resistant tuberculosis patients at University of Gondar Comprehensive Specialized Hospital, Northwest Ethiopia: a retrospective follow-up study

**DOI:** 10.1186/s12879-019-4447-8

**Published:** 2019-09-18

**Authors:** Getahun Molla Kassa, Alemayehu Shimeka Teferra, Haileab Fekadu Wolde, Atalay Goshu Muluneh, Mehari Woldemariam Merid

**Affiliations:** Department of Epidemiology and Biostatistics, Institute of Public Health, College of Medicine and Health Sciences and Specialized Comprehensive Hospital, University of Gondar, Gondar, Ethiopia

**Keywords:** Drug-resistant tuberculosis, Incidence, Predictors, Lost to follow-up, Retrospective study

## Abstract

**Background:**

The emergence of Drug-Resistance Tuberculosis (DR-TB) is an increasing global public health problem. Lost to Follow-up (LTFU) from DR-TB treatment remains a major barrier to tuberculosis epidemic control and better treatment outcome. In Ethiopia, evidences on the incidence and predictors of LTFU are scarce. Thus, this study aimed to determine the incidence and identify the predictors of LTFU among DR-TB patients.

**Methods:**

A retrospective follow-up study was conducted among a total of 332 DR-TB patients at the University of Gondar comprehensive specialized hospital. Data were retrieved from patient records from September 2010 to December 2017 and entered in to Epi-data 4.2.0.0 and analysed using Stata14.1 software. The risk was estimated using the Nelson-Aalen cumulative hazard curve. A log-rank test was used for survival comparisons between categories of independent variables. The Gompertz regression model was fitted, and hazard ratio with a 95% confidence interval (CI) was used to measure the strength of associations. Variables with less than 0.05 *p*-values in the multivariable model were considered as significantly associated with LTFU.

**Results:**

Among a total of 332 patient records reviewed, 206 (62.05%) were male. The median age was 30 years (Inter Quartile Range (IQR): 23–40). Forty-one (12.35%) of the participants had no history of TB treatment, while a quarter of were TB-HIV co-infected. Closely all (92.17%) of the patients had pulmonary tuberculosis. The median follow up time was 20.37 months (IQR: 11.02, 21.80). Thirty-six (10.84%) patients were lost from follow-up with an incidence rate of 6.47 (95% CI: 4.67, 8.97)/1000 Person Months (PM). Homelessness (Adjusted Hazard Ratio (AHR) =2.51, 95%CI: 1.15, 5.45) and treatment enrolment year from 2013 to 2014 (AHR = 3.25, 95% CI: 1.30, 8.13) were significant predictors of LTFU.

**Conclusion:**

This study indicated that LTFU among DR-TB registered patients was high in the first six months compared to subsequent months. Homelessness and year of treatment enrolment were independent predictors of LTFU, requiring more economic support to patients in order to ensure treatment completion. This result can be generalized to patients who are using DR-TB treatment in similar settings.

## Background

Tuberculosis (TB) is currently a significant public health problem worldwide [1]. The emergence of DR-TB, defined as resistance to at least one of the core first line anti-TB drugs, has become a threat to the global TB care and prevention [2]. According to the 2018 World Health Organization (WHO) TB report, an estimated 3.5% of the new and 18% of the previously treated TB cases had Multi-Drug Resistant (MDR) or Rifampicin Resistant (RR) TB, respectively [3]. Nearly 50 % of the global DR-TB cases were found in China, India, and the Russian Federation [2–5]. Worldwide, 600,000 and 558,000 DR-TB cases emerged in 2016 and 2017, respectively [3, 5].

In 2017, the estimated prevalence of MDR or RR TB in Africa was 2.7 and 14% among new and previously treated TB patients, respectively [3]. A meta-analysis in Sub-Saharan countries showed the pooled prevalence of DR-TB among the new and the previously treated patients was estimated to be 12.6 and 27.2%, respectively [6].

Ethiopia is one of the 30 high DR-TB burden countries globally and stands third among African countries with annual estimates of 2100 DR-TB cases from a total of annually notified TB cases [7]. Two DR-TB surveys in 2005 and 2014 and the 2018 WHO TB report in Ethiopia showed that the prevalence of DR-TB was 1.6, 2.3, and 2.7% among new TB cases, and 11.8, 17.8, and 14% among previously treated ones, respectively [3, 7, 8]. According to one meta-analysis, the overall prevalence of MDR-TB among newly diagnosed and previously treated TB patients was 2 and 15%, respectively [9]. DR-TB has been widely distributed throughout Ethiopia [8, 10].

Globally, TB incidence is falling by 2% annually; the fastest decline since 2010 has exceeded 4% per year in several high TB burden countries, including Ethiopia. However, to reach the first milestones of the “end TB strategy” by the year 2020, the annual reduction of TB incidence should be 4 to 5% [5].

Global DR-TB treatment success rate was nearly 50% although some high burden countries including Ethiopia showed a treatment success rate of more than 75% and this low treatment success rate has been due to high LTFU and deaths [2, 3, 11]. Lost to follow-up from DR-TB treatment remains a major barrier to the cure and epidemic control of DR-TB [12, 13].

According to a qualitative study done in India, developing short duration treatment regimens, reducing pill burden, and providing of motivational counseling [14] were the top identified themes that can reduce LTFU. Better general TB knowledge and higher levels of trust in clinical teams [15] were also protective factors against LTFU. Studies in Asia and Africa reported that adverse drug effects [14–16], ambulatory model of treatment initiation, and different providers in the intensive and continuation phase of treatment [17] were risk factors to LTFU.

The few studies done on treatment outcome and its determinants among patients with DR-TB in Ethiopia showed that poor treatment outcome was due to LTFU and death [18, 19]. But there has been only limited evidence on the incidence and predictors of LTFU among DR-TB treatment patients in the country; despite the high burden of DR-TB cases. Therefore, estimating the rate and identifying the risk factors for LTFU is essential to prioritize the high risk groups of patients for clinical and programmatic interventions, to achieve the Sustainable Development Goals (SDGs) and the end TB strategy targets, to prevent the community from primary DR-TB infection, and to reduce further drug resistance developments. In addition, this study will also inform the respective stakeholders about the current state of LTFU among DR-TB patients, and assist in planning.

## Methods

### Study design and setting

An institution-based retrospective follow-up study was conducted on DR-TB patients taking treatments at the University of Gondar Comprehensive Specialized Hospital from September 2010 to December 2017.

The hospital is located in Gondar city, Amhara National Regional State, 738 km to the northwest of Addis Ababa, the capital of Ethiopia. The University of Gondar comprehensive specialized teaching hospital serves more than seven million people of North Gondar and neighbouring zones. It is the second oldest hospital that started DR-TB treatment in September 2010 s only to St. Peter’s TB specialized hospital in the country and first in Amhara Region. Gondar has one treatment initiation and five treatment follow-up centres for DR-TB. There were also ten government and twelve public private mix DOTs clinics in the city. The hospital had two Xpert MTB/RIF assay machine, and external quality assured TB culture and acid fast staining sites for TB diagnosis. A total of 341 DR-TB patients were treated at the University of Gondar comprehensive specialized hospital from the beginning of the DR-TB treatment up to December 2017.

### Study population and data collection procedures

The source population was all patients enrolled at the hospital for DR-TB treatment, while the study population included all 341 new drug-resistant tuberculosis patients registered form September 2010 to December 2017.

The data were collected from patient follow-up charts, registration books, and computer data base using a data extraction checklist which was prepared in English. The records to be reviewed were identified using patient identification numbers. The data were collected by three bachelor degree graduate nurses working at the TB ward under a close supervision of the principal investigator. In order to control the quality of data, training was given to data collectors on the objectives of the study and on how to extract data from patient records. The supervisor checked the completeness and consistency of the extracted data daily.

### Variables

The outcome variable of this study was time in months from the start of treatment to LTFU or censored. Lost to follow-up was defined as a patient who took anti-TB treatment for any duration and interrupted it for two or more consecutive months for any reason; censored was defined when LTFU was not observed during the follow-up time which might include cured, treatment completed, treatment failed, treatment stopped, dead, transfer out and those who were still on treatment at the end of the study [7, 20] (Table 1).

**Table 1:** Definitions of treatment outcome for drug-resistant tuberculosis

Outcome	Definition
Cure	Treatment completed according to national recommendation without evidence of failure and three or more consecutive cultures taken at least 30 days apart are negative after the intensive phase.
Completed	Treatment completed according to national recommendation without evidence of failure but no record that three or more consecutive cultures taken at least 30 days apart are negative after the intensive phase.
Treatment failure	Treatment terminated or need for permanent regimen change of at least two anti-TB drugs because of lack of conversion by the end of the intensive phase, or bacteriological reversion in the continuation phase after conversion to negative after intensive phase, or evidence of additional acquired resistance to fluoroquinolones or second line injectable drugs, or Adverse drug reactions.
Died	A patient who dies for any reason during the course of TB treatment.
Not evaluated	A TB patient for whom no treatment outcome is assigned. This includes “transferred out” cases with unknown outcome at reporting unit.

Different characteristics at baseline were assessed from the registration document of the patients. The first characteristic assessed was socio-demographic which included age, sex, employment status, educational status, marital status, residence, region, and housing conditions. With regard to housing conditions, homeless was defined as patients who lived in streets or lacked fixed, regular, and adequate night-time residence. The second characteristics were behavioural components. These include smoking and alcohol drinking status. The third characteristics were clinical components, which included HIV co-infection, functional status, site of TB disease, baseline sputum smear microscopy result, culture conversion, presence of chronic complications, lung complication, DR-TB type, baseline BMI and TB treatment history. TB treatment history was classified as previously treated and untreated cases. Previously treated cases were defined as patients who were treated for TB for 1 month or more without considering chemoprophylaxis [7, 20]. The fourth characteristic considered was program related factor which included the year of enrolment.

### Data processing and analysis

After the data was checked for its consistency and completeness, it was entered in to epi-data version 4.2.0.0 and exported to Stata version 14.1 software for further cleaning and analysis. Summary statistics were carried out to describe demographic, behavioural, clinical, and programmatic data. Incidence rate was calculated by dividing the total number of LTFU to the total person months of observations. Life table was used to estimate cumulative failure at a different point in time. The Nelson-Aalen cumulative hazard curve was employed to estimate overall failure rate. The log-rank test was used to compare survival experience among different exposure groups. The best fitted model was selected based on the highest Likelihood (LL) and lowest information criteria (AIC and BIC), and the Gompertz regression model was fitted. The Cox proportional hazard assumption was tested for each variable and globally by using Schoenfeld residuals. The Nelson-Aalen cumulative hazard verses the Cox-Snell residual graph was used to test the goodness of the model fit. The bivariable model was fitted first and variables with a *p*-value less than or equal to 0.25 were used in the final multivariable model to identify the predictor variables. Finally, the Adjusted Hazard Ratio (AHR) with a 95% confidence interval (CI) in the multivariable model was used to select independent predictor variables of time to LTFU.

## Results

### Baseline socio-demographic and behavioural characteristics

A total of 341 patients who started treatment between 2010 and 2017 were eligible for this study of which 9 patients were excluded because five patient charts were lost and four patient outcomes were not evaluated. Therefore, 332 patient records were eligible for analysis; among these, 206(62.05%) of the patients were male. The median age of the total participants was 30 years (IQR: 23–40), and the median age for lost patients was 29 (IQR: 25–45) years. Three hundred twelve (93.98%) of the participants were from the Amhara Regional State. Nearly 40% (131) of the participants were married (Table 2).

**Table 2:** Baseline socio-demographic and behavioural characteristics of DR-TB patients stratified by lost to follow-up, University of Gondar Comprehensive Specialized Hospital, September 2010–December 2017, (*N* = 332)

Variable	Frequency (%)	Lost to follow-up Status	
		Event^a^ (*n* = 36)	Censored^b^ (*n* = 296)
Sex			
Male	206 (62.05)	20	186
Female	126 (37.95)	16	110
Residency			
Urban	169 (50.90)	19	150
Rural	163 (49.10)	17	146
Region			
Amhara	312 (93.98)	30	282
Tigray	19 (5.72)	6	13
Benshangul Gumuz	1 (0.30)	0	1
Marital status			
Married	131 (39.46)	18	113
Single	104 (31.33)	10	96
Divorced	36 (10.84)	4	32
Widowed	9 (2.71)	2	7
Separated	29 (8.74)	1	28
< 18 years	19 (5.72)	2	17
Not recorded	4 (1.20)	1	3
Level of education			
Not formally educated	120 (36.14)	16	104
Primary	104 (31.33)	11	93
Secondary	54 (16.27)	6	48
Tertiary	44 (13.25)	2	42
Not in school age	5 (1.51)	0	5
Not recorded	5 (1.51)	1	4
Housing condition			
Homeless	46 (13.85)	10	36
Having Home	248 (74.70)	26	222
Not recorded	38 (11.45)	0	38
Occupation			
Not employed	117 (35.24)	9	108
Employed	209 (62.95)	26	183
Not recorded	6 (1.81)	1	5
Smoking			
No	277 (83.43)	32	245
Yes	54 (16.27)	3	51
Not recorded	1 (0.30)	1	0
Alcohol drink			
No	279 (84.04)	30	249
Yes	50 (15.06)	5	45
Not recorded	3 (0.90)	1	2

Event^a^ in this study was patients lost during follow-up; Censored^b^ was either cured, completed, death, transfer out, still on treatment

**Fig. 1:**
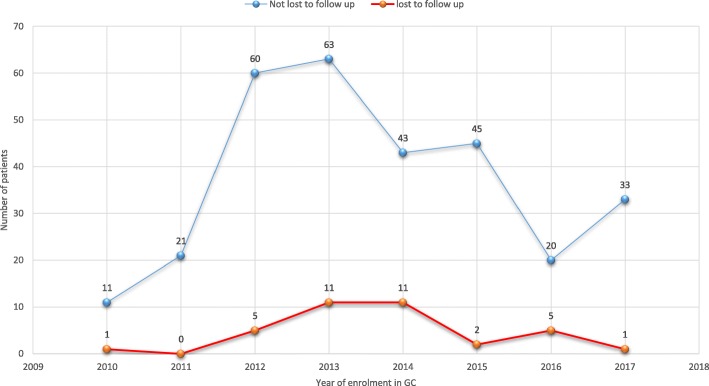
Trend of the number of DR-TB patients lost and not lost to follow up by year of treatment enrolment at University of Gondar Comprehensive Specialized Hospital, September 2010–December 2017

The total number of DR-TB patients and number of lost patients on treatment gradually increased from 2010 to 2013 and then declined unevenly (Fig. 1).

### Baseline clinical status of the participants

More than 67% (225) of the cases were ambulatory. Nearly 72% had a baseline body mass index of less than 18.5 kg/m^2^. A quarter of patients (84/330) that had test results for HIV were positive. Among those who had recorded results, closely all (92.17%) of patients had pulmonary tuberculosis with baseline smear and culture positivity of 84.31% (258/306) and 71.71% (180/251) respectively (Table 3).

**Table 3:** Baseline clinical characteristics of DR-TB patients stratified by lost to follow-up status, University of Gondar Comprehensive Specialized Hospital, September 2010–December 2017, (*N* = 332)

Variable	Frequency (%)	Lost to follow-up status	
		Event^a^ (*n* = 36)	Censored^b^ (*n* = 296)
BMI			
< 18.5	221 (71.99)	25	186
> =18.50	86 (18.01)	8	78
Functional status			
Working	78 (23.49)	7	71
Ambulatory	225 (67.77)	29	196
Bedridden	29 (8.73)	0	29
Registration Group			
New	41 (12.35)	4	37
Relapse	45 (13.55)	5	40
Return after lost to follow-up	2 (0.60)	1	1
Failure	241 (72.60)	26	215
outcome not assigned	3 (0.90)	0	3
No of previous TB treatment			
< 2	112 (33.74)	11	101
> =2	218 (65.66)	25	193
Not recorded	2 (0.60)	0	2
Site of tuberculosis			
Pulmonary	306 (92.17)	31	275
Extra Pulmonary^c^	26 (7.83)	5	21
Lung complication			
No complication	275 (82.83)	29	246
Pneumonia	39 (11.75)	5	34
Pneumothorax	5 (1.51)	0	5
Corpulmonale	9 (2.71)	1	8
Bronchiectasis	4 (1.20)	1	3
Co-morbidity			
No	277 (83.43)	30	247
Yes^d^	55 (16.57)	6	49
HIV co-infection			
No	246 (74.10)	27	219
Yes	84 (25.30)	9	75
Unknown	2 (0.60)	0	2
Base line sputum smear result			
Negative	48 (14.46)	6	42
Positive	258 (77.71)	27	231
Not recorded	26 (7.83)	3	23
Baseline Culture result			
Positive	180 (54.22)	15	165
Negative	71 (21.38)	12	59
Not recorded	81 (24.40)	9	72
Culture converted among baseline Culture result positive			
Yes	159 (88.33)	9	150
No	21 (11.67)	6	15
DR-TB type			
RR-TB	147 (44.28)	17	130
MDR-TB	168 (50.60)	18	150
Clinically diagnosed	17 (5.12)	1	16

Event^a^ in this study was patients lost during follow-up; Censored^b^ was either cured, completed, death, transfer out, still on treatment

^c^Lymph node, vertebral, bone, testicular, and skin

^d^Diabetes Mellitus, Hypertension, Bronchial Asthma, CKD, and Cardiac diseases

### Lost to follow-up of patients on DR-TB treatment

Patients were followed for a minimum of 0.33 and a maximum of 27.30 months with a total of 5564.67 person-months (PM) of observations. The median follow-up time was 20.37 months (IQR: 11.02–21.80). Regarding treatment outcome, 177 (53.31%) patients were cured, 35 (10.54%) completed treatment, 36 (10.84%) died, 36 (10.84%) LTFU, 40 (12.05%) on treatment, 2 (0.60%) transferred out, and 6 (1.81%) had treatment failure. The overall proportion of LTFU was 10.84% (95% CI: 7.91, 14.69). The proportions of LTFU among all lost patients were 22, 56, 67, and 81% at the end of the 3rd, 6th, 8th, and 10th months of follow-up, respectively.

The overall incidence rate of LTFU was 6.47 (95% CI: 4.67, 8.97) /1000 person-month (PM) observation. The incidence rate per 1000 person-month of observation was 105.39 [95% CI: 67.99, 163.36], 34.93 [95% CI: 18.79, 64.89], 9.65 [95% CI: 3.62, 25.72] and 0.48 [95% CI: 0.12, 1.94] in the first 6th, 7th–12th, 13th–18th, and 19th–24th months, respectively. Moreover, the incidence rates per 1000 person-months of observation were 3.20 [95% CI: 1.44, 7.12], 10.36 [95% CI: 6.82, 15.74], and 5.11 [95% CI: 2.55, 10.21] before the year 2013, from 2013 to 2014, and after 2014 respectively.

**Fig. 2:**
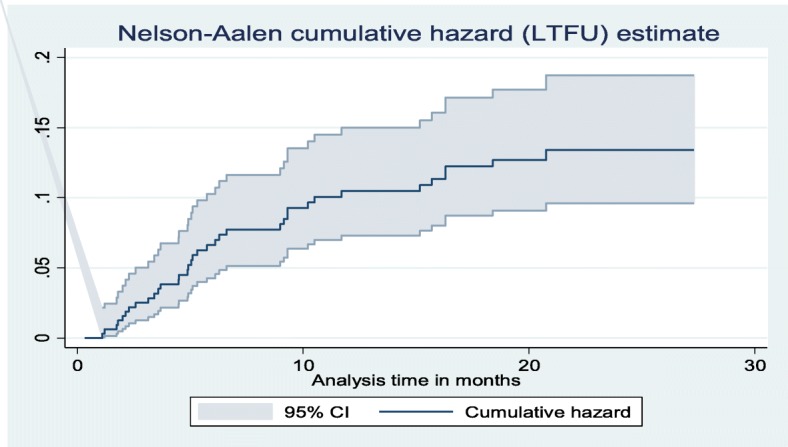
Nelson-Aalen cumulative hazard estimation of LTFU of patients under DR-TB Treatment at University of Gondar Comprehensive Specialized Hospital, September 2010–December 2017

The cumulative probability of LTFU was 6.37% (95% CI: 4.16, 9.70) at the 6th month, 9.90% (95% CI: 7.02, 13.87) 12th month, 11.45% (95% CI: 8.31, 15.68) 18th month, and 12.92% (95% CI: 9.37, 17.69) at the 24th month after the start of the follow-up (Fig. 2).

### Predictors of lost to follow-up

**Fig. 3:**
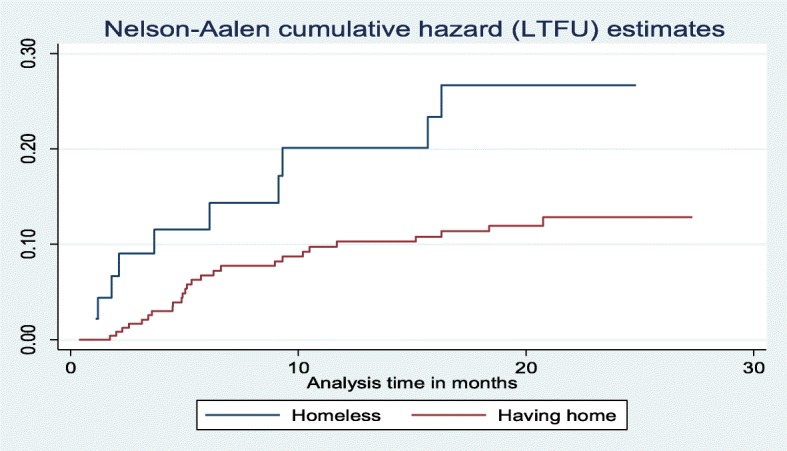
Nelson-Aalen cumulative hazard curve of lost to follow up by housing status on time to LTFU among DR-TB patients at University of Gondar Comprehensive Specialized Hospital, September 2010–December 2017

**Fig. 4:**
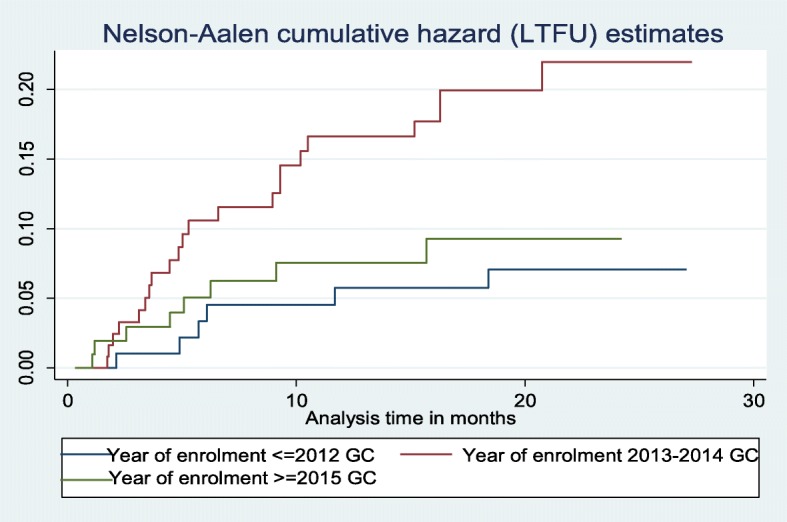
Nelson-Aalen cumulative hazard curve of lost to follow up by year of treatment start on time to LTFU among DR-TB patients at University of Gondar Comprehensive Specialized Hospital, September 2010–December 2017

A log-rank test (X^2^) of equality of hazard of incidence of LTFU was done for the different categories of explanatory variables. The test result indicated a significant difference in the incidence of lost to follow-up between patients who were homeless and who had home, *p*-value = 0.0046, and patents who were enrolled before 2013, from 2013 to 2014 and after 2014, *p*-value = 0.0152 at 5% level of significance (Figs. 3 and 4).

The proportional-hazards assumption was assessed based on the Schoenfeld residuals, and it was found that all of the covariates and the full model satisfied the proportional hazard assumption with the global test, *p*-value = 0.8423.

According to the information criteria (AIC, BIC) and LL comparison techniques, the Gompertz regression model was found to be more efficient than the other semi-parametric and parametric models fitted (Table 4).

**Table 4:** Summary of model comparison between Cox proportional hazard model and parametric survival distribution models using likelihood (LL) and information criteria (AIC and BIC)

Comparison Method	Models				
	*Cox PH*	*Weibull*	*Exponential*	*Gompertz* ^a^	*Log logistic*
LL	− 183.4165	− 139.9643	− 140.8364	− 136.894	− 139.3641
AIC	378.833	295.9287	295.6728	289.7879	294.7281
BIC	400.8316	325.2601	321.3378	319.1193	324.0596

^a^Highest LL and lowest AIC and BIC

In the bivariable Gompertz regression analysis, age, housing condition, treatment start year, cigarette smoking, and occupation were associated with lost to follow-up. However, in the multivariable Gompertz regression analysis, housing condition and treatment start year remained statistically significant predictors of LTFU.

The risk of lost to follow-up among homeless patients was two and half times at higher hazard of LTFU compared to those who had home (AHR = 2.51, 95%CI: 1.15, 5.45). Patients who started treatment in the year 2013 and 2014 were more than three times at higher hazard of lost to follow-up than those who started treatment before 2013 (AHR = 3.25, 95% CI: 1.30, 8.13) (Table 5).

**Table 5:** Bivariable and multivariable Gompertz regression analysis for predictors of time to lost to follow-up among DR-TB patients, University of Gondar Comprehensive Specialized Hospital, September 2010–December 2017, (*N* = 332)

Variable	Event^a^	Censored^b^	CHR (95%CI)	AHR (95%CI)
Median Age (IQR)	29 (25, 45)	30 (22, 40)	1.02 (1.00, 1.05)	1.03 (0.99, 1.05)
Sex				
Male	20	186	1	
Female	16	110	1.34 (0.69, 2.58)	
Body mass index (kg/m^2^)				
< 18.5	25	196	1.22 (0.55 2.71)	
> =18.50	8	78	1	
Housing Condition^c^				
Homeless	10	36	2.20 (1.06, 4.57)	2.51 (1.15, 5.45)
Having Home	26	222	1	1
Alcohol drink				
No	30	249	1	
Yes	5	45	0.91 (0.35, 2.35)	
Co-morbidity				
No	30	247	1	
Yes	6	49	1.12 (0.46, 2.68)	
Year of treatment initiation^c^				
< =2012	6	92	1	1
2013–2014	22	106	3.08 (1.25, 7.59)	3.25 (1.30, 8.13)
> =2015	8	98	1.42 (0.49, 4.11)	2.07 (0.71, 5.99)
Base line sputum smear result				
Negative	6	42	1	
Positive	27	231	0.77 (0.32, 1.87)	
Residency				
Urban	19	150	1	
Rural	17	146	0.97 (0.51, 1.86)	
Cigarette smoking				
No	32	245	1	
Yes	3	51	0.47 (0.14, 1.53)	0.40 (0.12, 1.35)
HIV co-infection				
No	27	219	1	
Yes	9	75	1.05 (0.49, 2.24)	
Number of previous TB treatment				
< 2	11	101	1	
> =2	25	193	1.10 (0.54, 2.24)	
Occupation				
Employed	26	183	1	
Unemployed	9	108	0.62 (0.29, 1.32)	0.74 (0.32, 1.68)

LR chi^2^ (6) = 17.14 probability > chi^2^ = 0.0088

*CHR* Crude Hazard ratio, *AHR* Adjusted Hazard Ratio, *CI* Confidence Interval

Event^a^ in this study was patients lost during follow-up; Censored^b^ was either cured, completed, death, transfer out, still on treatment

The shape parameter gamma was found to be −0.0811245 (95% CI: −0.1407007, −0.0215482) which is negative. This indicates that the hazard of lost to follow-up decreases exponentially with time

^c^Significantly associated variables in both bivariable and multivariable model

**Fig. 5:**
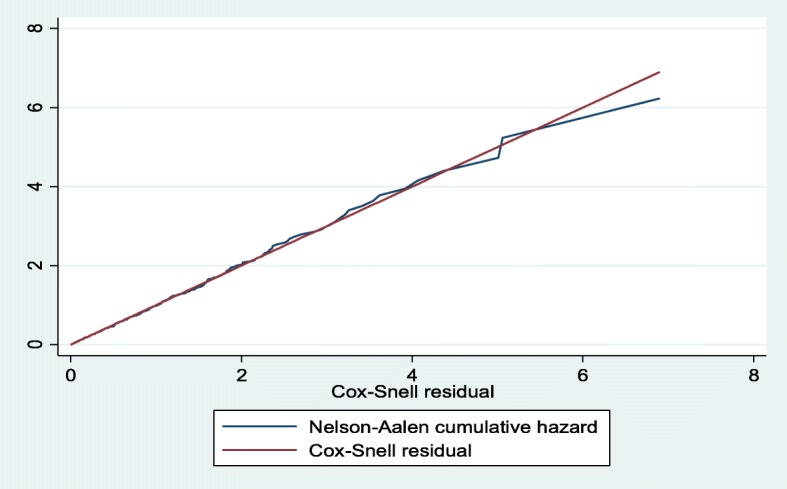
Nelson-Aalen cumulative hazard against Cox-Snell residual plot for Gompertz model for LTFU among DR-TB patients at University of Gondar Comprehensive Specialized Hospital, September 2010 – December 2017

Goodness of fit for the selected model was assessed using the Cox-Snell residual test, and the result showed the model provided the best fit for our data (Fig. 5).

## Discussion

This study was designed to assess the incidence rate and to identify the predictors of time to lost to follow-up among DR-TB patients attending their treatment at the University of Gondar Comprehensive Specialized Hospital. The incidence rate of lost to follow-up was found to be 6.47 with 95% CI: [4.67, 8.97] per 1000 PM of observation, i.e. if we followed 1000 DR-TB patients for a month during their treatment, 6.47 patients were lost from follow-up at the end of the month. The incidence rate per 1000 PM of observation in every 6 month interval (the first 6, 7–12, 13–18, and 19–24) was 105.39, 34.93, 9.65 and 0.48 respectively, i.e. incidence rate of LTFU was higher in the first 6 months compared to subsequent follow-up months. This finding showed that the incidence rate of LTFU was a challenge to end the epidemic of TB targeted by SDGs and the End TB strategy by 2030 and 2035, respectively [13, 21] in the study area and similar settings. From the total lost to follow-up patients, 22.2% (8/36) and 55.6% (20/36) were lost in the first three and 6 months of treatment, respectively. This finding was low compared to a finding from India which showed more than half were lost in the third [22] and 86.9% in the sixth [17] months of treatment. On the other, hand the finding was higher compared to that of a study in South Africa which showed 8.19% of the patients were lost in the third month of treatment [23]. These differences might be due to differences in the mode of treatment delivery in that the study from India was based on the ambulatory model of care (without initial hospitalization), and the possible reason for the high lost in the South African study might be the use of the community level study design. In general, the likely explanation for patients to be lost in the early period of treatment might be due to adverse drug effects commonly associated with injectable drugs mostly taken during the first phase of treatment [14, 16, 24]. This could make exhaust and urge patients to interrupt their treatments due to repeated treatments with first line anti-TB drugs which in turn leads patients to be lost from follow-up [14, 16, 24]. Among lost patients, 86% were pulmonary TB cases, and out of those who had data on culture result at the time of lost to follow-up, 40% were not culture converted. This finding was similar to a finding in a Georgian study (40%) [24]. Patients with positive culture result are very infectious and puts communities at risk for developing primary DR-TB. The above findings illustrate the challenges to achieving completion of the recommended regimens for DR-TB and the TB prevention strategy at that movement [14, 24]. Evidence from different high DR-TB burden countries noted that low default rates or good DR-TB treatment success rates were related to the implementation of community-based treatment practices which were not yet implemented in Ethiopia. A community-based treatment practice is an effective strategy for increasing access to treatment which is patient centered and more acceptable to patients in that it has a great contribution to the retention of patients in the care [23, 25, 26].

In this study, we tried to assess factors which predicted the time to LTFU and noted that homelessness and year of treatment enrolment remained independent predictors for lost LTFU.

The hazard of lost to follow-up among homeless patients was two and half times higher compared to those who had home. Similar association were reported by studies in rural South Africa [12], Limu Peru [27], and one meta-analysis [28]. Despite the provision of individualized socioeconomic support to all patients by the national Programmatic Management of Drug-resistant Tuberculosis (PMDT) program, LTFU among homeless patients was high. This might be explained by the fact that homelessness is extremely linked to poverty that triggers the risk for LTFU [29]. Another reason could be the difficulty of tracing such patients when they interrupt treatment. Moreover, it can also be explained in that majority (67.47%) of our patients were in the age of thirties who might be responsible to support their families that could trigger to lost from follow-up. The relationship between financial constraints, adequate nutrition, and the fear of losing jobs were challenges to patients who need to complete treatments as evidenced by different studies [14, 29]. Therefore, the disparity between working hours and treatment center schedules as well as inability to work due to treatment side effects might be the possible reasons for the higher hazard of LTFU among homeless patients.

Patients who started treatment in the years 2013 to 2014 were more than three times at higher hazard of lost to follow-up compared to those who started treatment before 2013. This finding was supported by those of studies done in Georgia [24] and Limu, Peru [27]. This can be justified by the fact that in 2013 to 2014 years of treatment enrolments the rise in the number of LTFU was thought to be secondary in light of the decreasing financial support and the need to spreading the money over the increasing number of patients. In addition, it was also believed to be secondary to the decreasing ability to provide individualised patient-centred care as the total DR-TB cohort size increased. Although previous studies showed that decentralized care appeared to be more likely to decreased lost to follow-up [26, 30], during the year 2013 to 2014 the Ethiopian National Tuberculosis Control Program (NTP) implemented a new PMDT guideline that promoted the decentralization of DR-TB services, and patients were linked to the newly established treatment initiating centers in the region. During linkage to the new sites, poor patient preparation and unequally equipped new sites might have increased the number of lost patients. The medical staff at that time witnessed that during the decentralization patients were linked from highly equipped, multi-disciplinary, more experienced, and more socioeconomically supporting site, to less experienced, new sites which dissatisfied patients by the services. Furthermore, the clinical team also had chances of visiting the new DR-TB TICs and communicating with some of the lost patients and confirmed that the newly developed treatment sites were primary hospitals with lots of shortcomings compared to the University of Gondar hospital. The University of Gondar Hospital DR-TB treatment site team were composed of multi-discipline teams (physicians, nurses, social workers, psychiatrist, expert patients, and other supporting non-governmental organizations like Global Health Committee).

Lost to follow-up in DR TB patients is an important routine indicator of the achievement of better treatment outcomes. Preventing lost from DR-TB treatment can improve patient intended treatment outcome, future health status, and prevent further drug resistant strains of tuberculosis. In addition, it is vital in TB infection control strategy since patients with DR-TB and positive sputum result are infectious and may transmit the disease to the people at risk in the general community. Despite the greet efforts we made to estimate the incidence and identify the predictors of LTFU among DR-TB patients our study has some limitations, since we used secondary data, some important socio-demographic, bacteriological, and economic factors which might have effects on LTFU could have been missed. Besides, the small number of patients still on treatment at the end of study who have the chance of LTFU might have under estimated the incidence.

## Conclusion

This study found that lost to follow-up among DR-TB registered patients for treatment was high in the first 6 months compared to later follow-up months. Housing status and year of treatment enrolment were independent predictors of time to LTFU. This result could be generalized to patients who are using DR-TB treatment in a similar setting in Ethiopia. There is a need for screening and prioritising risk factors and developing programmatic strategies to provide comprehensive socioeconomic support (incentives and/or enablers) to socioeconomically underprivileged patients in order to ensure treatment success.

## Data Availability

The datasets used during the current study are available from the corresponding authors upon reasonable request and with permission of University of Gondar Comprehensive Specialized Hospital.

## Abbreviations

DR-TB: Drug Resistance Tuberculosis
LTFU: Lost To Follow-up
MDR-TB: Multi-Drug Resistant Tuberculosis
PM: Person Months
PMDT: Programmatic Management of Drug-resistant Tuberculosis
RR-TB: Rifampicin Resistance Tuberculosis
SDGs: Sustainable Development Goals
TB: Tuberculosis
TIC: Treatment Initiation Center
WHO: World Health Organization
